# Structure-guided selection of specificity determining positions in the human Kinome

**DOI:** 10.1186/s12864-016-2790-3

**Published:** 2016-08-18

**Authors:** Mark Moll, Paul W. Finn, Lydia E. Kavraki

**Affiliations:** 1 Department of Computer Science, Rice University, PO Box 1892, Houston, 77251 TX USA; 2 University of Buckingham, Hunter St, Buckingham, UK

**Keywords:** Protein kinases, Specificity determining positions, Binding affinity

## Abstract

**Background:**

The human kinome contains many important drug targets. It is well-known that inhibitors of protein kinases bind with very different selectivity profiles. This is also the case for inhibitors of many other protein families. The increased availability of protein 3D structures has provided much information on the structural variation within a given protein family. However, the relationship between structural variations and binding specificity is complex and incompletely understood. We have developed a structural bioinformatics approach which provides an analysis of key determinants of binding selectivity as a tool to enhance the rational design of drugs with a specific selectivity profile.

**Results:**

We propose a greedy algorithm that computes a subset of residue positions in a multiple sequence alignment such that structural and chemical variation in those positions helps explain known binding affinities. By providing this information, the main purpose of the algorithm is to provide experimentalists with possible insights into how the selectivity profile of certain inhibitors is achieved, which is useful for lead optimization. In addition, the algorithm can also be used to predict binding affinities for structures whose affinity for a given inhibitor is unknown. The algorithm’s performance is demonstrated using an extensive dataset for the human kinome.

**Conclusion:**

We show that the binding affinity of 38 different kinase inhibitors can be explained with consistently high precision and accuracy using the variation of at most six residue positions in the kinome binding site. We show for several inhibitors that we are able to identify residues that are known to be functionally important.

## Background

Predicting affinity profiles remains a challenging task for computational and medicinal chemists. This is particularly true of the kinase family of enzymes because of their large number and structural similarity. Despite their structural similarity, the kinases exhibit large phylogenetic diversity. As a result, binding site sequence dissimilarity alone cannot explain the differences in binding affinity [1]. Selectivity patterns obtained by experimental screening in enzyme assays are often difficult to rationalize in structural terms. Additional tools are needed to improve our capabilities to design inhibitors that *selectively* bind to only a small subset of the kinases. The rapidly increasing number of kinase structures has made it possible to study how structural differences affect binding affinity. For instance, different inhibitors have been designed to target the inactive, DFG-out conformation and active, DFG-in conformation [2–5]. In general, determining exactly how functional changes relate to structural ones remains an important open challenge [6, 7]. This is caused in part by the fact that not all structural changes cause a functional change. Additionally, the available structures are non-uniformly distributed over the known kinase sequences: for many kinases there is no structural information, while other kinases are overrepresented, which can lead to overfitting.

In previous work [1], we introduced the Combinatorial Clustering Of Residue Position Subsets (CCORPS) method and demonstrated that it could be used to predict binding affinity of kinases. CCORPS considers structural and chemical variation among all triplets of binding site residues and identifies patterns that are predictive for some externally provided labeling. The labeling can correspond to, e.g., binding affinity, Enzyme Commission classification, or Gene Ontology terms, and only needs to be defined for *some* of the structures. CCORPS corrects for the non-uniform distribution of structures. From the patterns CCORPS identifies, multiple predictions are combined into a single consensus prediction by training a Support Vector Machine. A limitation of this work is that it is difficult to identify the most important Specificity Determining Positions (SDPs). In this paper, we are not trying to construct a better predictor, but, rather, a better explanation for some labeling. The explanation is better in the sense that it provides a simple explanation of a labeling in terms of the dominant SDPs. Rather than using *all* patterns discovered by CCORPS, it uses a small number of patterns that involve only a small number of residues yet is able to accurately recover binding affinity.

The main contribution of this paper is an algorithm that computes the Specificity Determining Positions that best explain binding affinity in terms of structural and chemical variation. More generally, the algorithm can identify a sparse pattern of structural and chemical variation that corresponds to an externally provided labeling of structures. This work extends our prior work on CCORPS, but shifts the focus from optimal predictions to concise, biologically meaningful, explanations of functional variation.

There has been much work on the identification and characterization of functional sites. Most of the techniques are broadly applicable to many protein families, but we will focus in particular on their application to kinases, when possible.

Much of the work on computing SDPs is based on evolutionary conservation in multiple sequence alignments (see, e.g., [8–10]). There has also been work on relating mutations to an externally provided functional classification in a phylogeny-independent way [11, 12]. This work is similar in spirit to what CCORPS does, but based on sequence alone.

While sequence alignment techniques can reveal functionally important residues in kinases [13], structural information can provide additional insights. This is especially true for large, phylogenetically diverse families such as the kinases. The FEATURE framework [14, 15] represents a radically different way of identifying functional sites. Instead of alignment, FEATURE builds up a statistical model of the spatial distribution of physicochemical features around a site.

Another approach to modeling functional sites has been the comparison of binding site cavities [3, 16]. In [17] a functional classification of kinase binding sites is proposed based on a combination of geometric hashing and clustering. This approach is similar in spirit to our prior work [1], but our work considers variations in a small sets of binding site residues, which makes it possible to separate non-functional structural changes from functional ones.

In [18] a method called FLORA is proposed for analysis of structural conservation across whole domains (rather than binding sites). FLORA was shown to be able to identify functional subfamilies (defined by Enzyme Commission classifications) within large protein superfamilies. It relies on the construction of structural feature vectors, which shares some similarities with our approach. However, FLORA is completely unsupervised and it is not clear how it could be extended to explain patterns of kinase binding affinity.

In [19] many of the ideas above are combined into one framework. Given sequences from a PFAM alignment [20] and some reference structures, homology models are constructed for all sequences. Next, cavities are extracted, aligned, and clustered. Unlike our work, the approach in [19] is completely unsupervised and does not aim to provide an explanation for an externally provided classification (such as kinase binding affinity).

## Methods

### CCORPS overview

Our algorithm builds on the existing CCORPS framework [1]. CCORPS is a semi-supervised technique that takes as input a set of partially labeled structures and produces as output the predicted labels for the unlabeled structures. Of course, this is only possible if the labels can be related to variations in the structures. In previous work [1] we have shown this to be the case for labelings based on binding affinity and functional categorization (Enzyme Commission classification).

CCORPS [1] consists of several steps. First, a one-to-one correspondence needs to be established between relevant residues (e.g., binding site residues) among all structures. This correspondence can be computed using a multiple sequence alignment or using sequence independent methods [21–24]. Second, we consider the structural and physicochemical variation among all structures and all triplets of residues. The triplets are not necessarily consecutive in the protein sequence and can be anywhere in the binding site. Each triplet of residues constitutes a *substructure*: a spatial arrangement of residues. For each triplet, we compute a distance matrix of all pairwise distances between substructures. The distance measure used is a combination of structural distance and chemical dissimilarity introduced in [22]. In particular, the distance between any two substructures *s*_1_ and *s*_2_ is defined as:

$${\begin{aligned} d(s_{1}, s_{2}) =& \ d_{\text{side chain centroid}}(s_{1}, s_{2}) + d_{\text{size}}(s_{1}, s_{2})\\ &+ d_{\text{aliphaticity}}(s_{1}, s_{2}) + d_{\text{aromaticity}}(s_{1}, s_{2})\\ &+ d_{\text{hydrophobicity}}(s_{1}, s_{2}) + d_{\text{hbond acceptor}}(s_{1}, s_{2})\\ &+ d_{\text{hbond donor}}(s_{1}, s_{2}). \end{aligned}} $$

The *d*_side chain centroid_(*s*_1_,*s*_2_) term is the least root-mean-square deviation of the pairwise-aligned side chain centroids of the substructures. The remaining terms account for differences in the amino acid properties between the substructures *s*_1_ and *s*_2_ as quantified by the pharmacophore feature dissimilarity matrix as defined in [22].

Each row in the distance matrix can be thought of as a “feature vector” that describes how a structure differs from all others with respect to a particular substructure. The *n*×*n* distance matrix for *n* structures is highly redundant and we have shown that the same information can be preserved in a 2-dimensional embedding computed using Principal Component Analysis [25]. Each 2D point is then a reduced feature vector. The set of *n* 2-dimensional points is clustered using Gaussian Mixture Models in order to identify patterns of structural variation. Not all structural variation is relevant; we focus on patterns of structural variation that align with the classification provided by the labeling.

The final stage of CCORPS is the prediction of labels for the unlabeled structures. Suppose a cluster for one of the residue triplets contains structures with only one type of label as well as some unlabeled structures. This would suggest that the predicted label for the unlabeled structures should be the same as for the other cluster members. We call such a cluster a *Highly Predictive Cluster* (HPC). These HPC are a critical component of the algorithm presented in the next section. There are many clusterings and each clustering can contain several HPCs (or none at all). For example, in the human kinome the binding site consists of 27 residues, leading to ${27\choose 3} = 2,925$ clusterings. Typically, an unlabeled structure belongs to several HPCs and we thus obtain multiple predictions. These predictions might not agree with each other. In our prior work we trained a Support Vector Machine [26] to obtain the best consensus prediction from the multiple predictions.

### Structure-guided selection of specificity determining positions

While CCORPS has been demonstrated to make accurate predictions, it has been difficult to interpret the structural basis for these predictions. This has motivated us to look at alternative ways to interpret the clusterings produced by CCORPS. Rather than trying to build a better predictor, we have developed an algorithm that constructs a *concise structural explanation* of a labeling. It determines a set of Specificity Determining Positions (SDPs). An algorithm that would predict that almost every residue position is important would not be very helpful. We therefore wish to enforce a sparsity constraint: for a set of labeled structures *S* we want to find the smallest possible number of HPCs that cover the largest possible subset of *S* and involve at most *λ* residues.

The problem of finding SDPs can be formulated as a variant of the set cover problem. The set cover problem is defined as follows: given a set *S* and subsets $S_{i}\subseteq S, i=1,\ldots,n$, what is the smallest number of subsets such that their union covers *S*? This is a well-known NP-Complete problem, but the greedy algorithm that iteratively selects the subset that expands coverage the most can efficiently find a solution with an approximation factor of $\ln |S|$.

As mentioned above, in our case, *S* is the set of *labeled* structures. We keep track of the residues involved in the selected HPCs and mark them as SDPs. Solving this as a set cover problem would likely still select most residues. The intuition for this can be understood as follows. The number of clusterings each residue is involved in is quadratic in the number of residues in the alignment. Each of those clusterings could contain a HPC that covers at least one structure that is not covered yet by other HPCs. Even in completely random data some patterns will appear, which could in turn be classified as HPCs.

We measure sparsity of the cover in terms of the number of residues and not the number of HPCs, since this facilitates an easier interpretation of the results shown later on. As noted before, there can be several HPCs per clustering. This means that once we have selected an HPC, we might as well include all other HPCs from that same clustering (we have already “paid” for using the corresponding residues). As an algorithmic refinement, we may also wish to limit the degree at which we are fitting the data to avoid overfitting and get a simpler description of the *most significant* residues positions whose variation can be used to explain the labeling.

The algorithm for computing SDPs is shown in Algorithm 1. It is similar to the greedy set cover algorithm. The input to the algorithm consists of a list of labeled structures, a list of all 3-residue subsets of the binding site, and a list of sets of structures that belong to HPCs. After initializing the set of SDPs and the set of selected subset indices in *S*, the main loop performs the following steps. First, the indices of all subsets are computed that will not grow the set of SDPs beyond a size limit *λ* (line 5). Second, the subset index is computed that will increase the cover of the known labels with HPC structures the most (line 9). Next, the algorithm checks whether the increase is “large enough,” i.e., greater than or equal to *δ* (line 11). If so, the set of SDPs and the sets of not-yet-covered structures are updated (line 13–14). If not, the algorithm terminates and returns the set of SDPs.



The final output of Algorithm 1 provides a concise explanation of which structural and chemical variations correlate highly with a given labeling. In the context of the kinases, it can identify triplets of residues whose combined structural and chemical variation give rise to patterns that allow one to separate binding from non-binding kinases. As we will see in the next section, often only a very small set of residues is sufficient to obtain HPCs that cover most of the structures with known binding affinity.

## Results

In [27] a quantitative analysis is presented of 317 different kinases and 38 kinase inhibitors. For every combination of a kinase and an inhibitor, the binding affinity was experimentally determined. This dataset also formed the basis for the evaluation of CCORPS [1]. The kinase inhibitors vary widely in their selectivity. Inhibitors like Staurosporine bind to almost every kinase, while others like Lapatnib bind to a very specific subtree in the human kinase dendrogram. The structure dataset was obtained by selecting all structures from the Pkinase and Pkinase_Tyr PFAM alignments [20]. The binding site, as defined in [1], consists of 27 residues. After filtering out structures that had gaps in the binding site alignment, 1,958 structures remained. The binding affinity values were divided into two categories (i.e., labels): “binds” and “does not bind.” This gives rise to two different types of HPCs: clusters predictive for binding (which we call true-HPCs below) and clusters predictive for *not* binding (which we call false-HPCs below). All other structures corresponding to kinases that were not part of the Karaman et al. study [27] do not have a label. CCORPS was run on this dataset, consisting of all 1,958 structures along with the binding affinity data. This resulted in ${27\choose 3}=2,925$ clusterings, one for every triplet of residues. The median number of true-HPCs per inhibitor was 591, while the median number of false-HPCs per inhibitor was 13,632.

In the next subsection we look in detail at results of our algorithm with one parameter setting to get a sense of what kind of output is produced. In the subsequent subsection we will describe different ways to measure coverage of the SDPs as well as their predictive potential. We then evaluate these measures on all inhibitors with different parameter settings.

### Specificity-determining positions

**Fig. 1:**
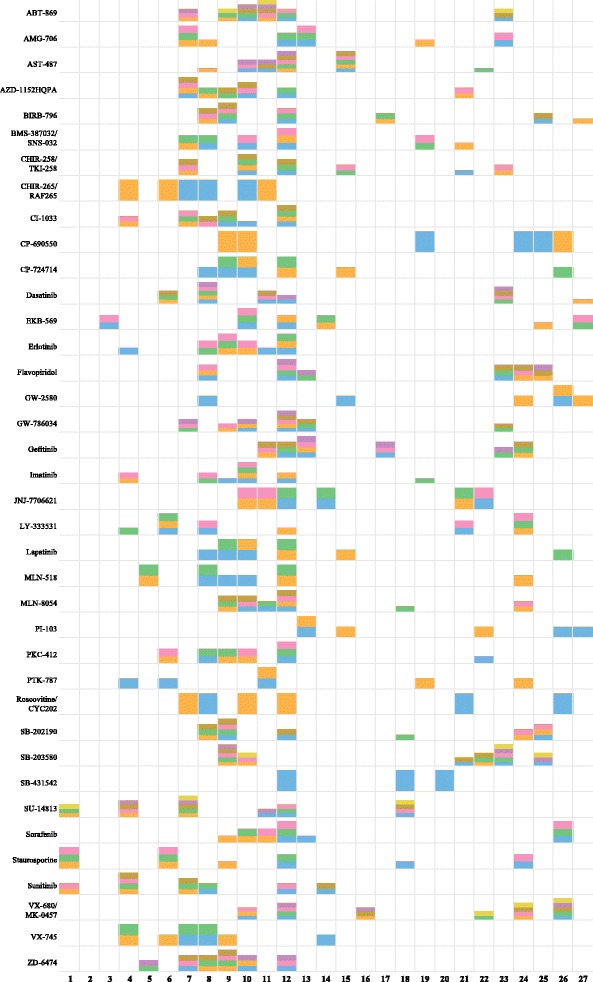
The SDP profiles computed for every inhibitor in the kinome dataset. The *x*-axis represents the residue position in the 27-residue multiple sequence alignment of the binding site. Each row shows the SDPs for one inhibitor whose name is shown on the *y*-axis. For each inhibitor, blocks with the same color correspond to one of the 3-residue subsets. If there are multiple colors in a given position, then the same residue was part of several selected subsets. This means that the same residue in different structural contexts can help explain the binding affinity of different kinases

While in our prior work [1] the emphasis was on *predicting* the affinity of kinases, here we are focused on creating a *concise explanation* of the affinity. Thus, here we are not performing cross validation experiments. We have run Algorithm 1 on the kinome dataset with *λ*=6 residues and *δ*=16 (statistics for different values of *λ* and *δ* are reported in the next subsection). With *λ*=6, the algorithm can select at most two non-overlapping triplets. We computed the SDPs for all inhibitors (see Fig. 1). With some additional bookkeeping we can keep track of which residue was involved in which selected subsets. The bar chart for each inhibitor can be interpreted as follows. Along the *x*-axis is the residue position in the multiple sequence alignment of the 27 binding site residues. The relative height of each bar indicates how often a residue position was part of a selected 3-residue subset. Blocks with the same color correspond to residues belonging to the same residue subset. This can provide important contextual information. It shows not only *which* residues are important to help explain binding affinity, but also the context in which its variation should be seen. It could, e.g., indicate that one residue’s variation *relative to* some other residue(s) is important. The contextual residues themselves may not always vary much and are perhaps not of as much functional importance in the traditional sense. As *λ* is increased, more bars would be added to each profile as long as they improve coverage by at least *δ* structures. Similarly, as *δ* is decreased, more bars would be added to each profile as long as no more than *λ* residues are involved.

**Fig. 2:**
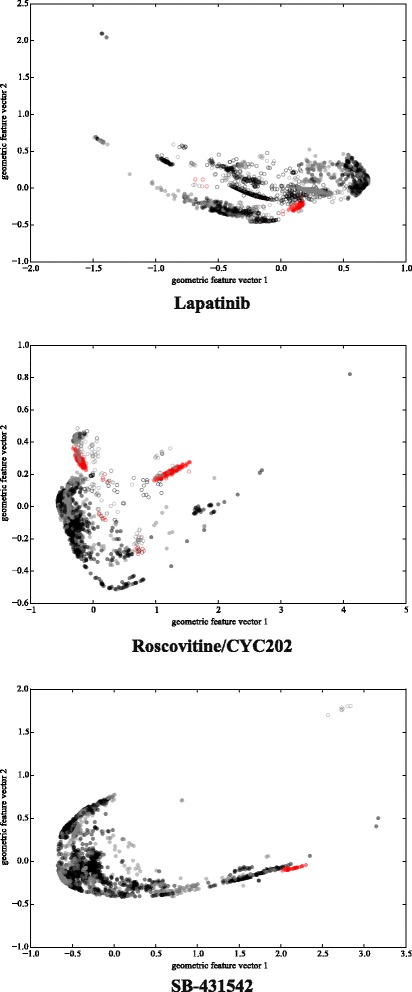
Examples of the kind of clusterings selected by our algorithm. The axes correspond to the 2D, PCA-reduced feature vector representation of the pairwise distances between structures as described in the Methods section. Each point represents one structure. *Red*: known to bind, *black*: known to not bind, *gray*: binding affinity unknown. *Discs*: structures belonging to HPCs, *circles*: all other structures

Figure 2 shows some examples of the clusterings that have been selected by Algorithm 1. These clusterings contain a large number of structures belonging to HPCs. The distance between points represents how different the corresponding structures are, structurally and chemically. The examples show that we can identify very strong spatial cohesion among the structures that bind when looking at the right residues (i.e., the SDPs). Not all clusterings selected by Algorithm 1 show such a strong relationship between structure and function. Especially for inhibitors that bind more broadly to kinases this relationship is harder to untangle.

There is significant variation among the SDP profiles. For a very selective inhibitor like SB-431542 the variation of only three positions is sufficient to explain the binding affinity (see also the next subsection), while for ABT-869 many combinations of 3 residues out of the 6 selected residues seem to be helpful in explaining the binding affinity.

**Fig. 3:**
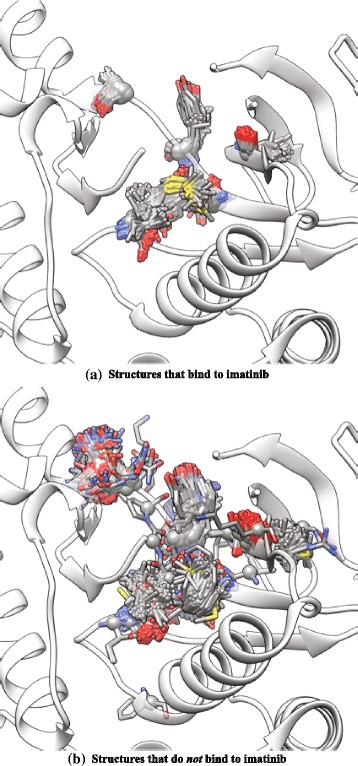
Structural visualization of SDPs. P38 (PDB ID 3HEC) is shown in ribbon representation along with the superimposed (**a**) SDPs for all the structures that bind imatinib and (**b**) the same residue positions for all structures that do *not* bind to imatinib

Figure 3 shows a visualization of the SDPs for the inhibitor Imatinib. Figure 3a shows the structural variation (or lack thereof) in the selected residue positions for all structures that bind Imatinib. In contrast, if the same positions in all structures that do *not* bind Imatinib are superimposed, the structural variation is very high as is shown in Fig. 3b.

### Coverage and predictive power of SDPs

Based on the set of SDPs we can (a) try to “recover” the labels of labeled structures that were not part of the selected HPCs and (b) predict labels for the unlabeled structures. There are at least four simple strategies to do this:

1. We could assume that the union of all true-hpcs contains all the structures that bind and that all others do not bind.
2. We could assume that the union of all false-hpcs contains all the structures that do not bind and all others do bind.
3. We could omit the false-hpcs altogether from the input *H* to Algorithm 1 and select residue subsets based on large true-hpcs only. The labels are then recovered as in (1).
4. We could omit the true-hpcs altogether from the input *H* to Algorithm 1 and select residue subsets based on large false-hpcs only. The labels are then recovered as in (2).

Note that the SDPs computed with Algorithm 1 are the same in the first two strategies, but will generally look different when using strategies 3 and 4. We have evaluated each of these strategies on all 38 ligands. For each we can evaluate the coverage: the percentage of known labels that are included in the HPCs. We can also count the number of *unlabeled* structures included in HPCs, which can be interpreted as the number of new binding affinities we can predict. For the first two strategies we get predictions for both binding and not-binding, while for the latter two we only get predictions for one type of affinity. Finally, we can calculate the usual statistical performance measures (sensitivity, specificity, precision, and accuracy) to measure how well the selected HPCs can predict binding affinity for all labeled structures. The results were computed with *λ*=6 and *δ*=16 and are summarized in Table 1. Note that specificity is equal to 1 in strategies 1 and 3 by construction. Similarly, sensitivity is equal to 1 in strategies 2 and 4 by construction. In general, assuming that the union of all true-HPCs contains all the structures that bind (as is done in strategies 1 and 3) results in poor sensitivity. Strategy 2 seems to strike a good balance between sensitivity and specificity as well as between precision and accuracy. Strategy 4 performs even better than strategy 2, but provides poorer coverage.

**Table 1:** Coverage of labeled structures, number of predicted affinities for unlabeled structures, as well as sensitivity, specificity, precision, and accuracy for HPC-based prediction of binding affinity

Strategy	Cov.	#pred.	Sens.	Spec.	Prec.	Acc.
1	83 %	215	0.486	1.000	0.921	0.904
2	83 %	520	1.000	0.887	0.783	0.929
3	15 %	1,084	0.617	1.000	0.921	0.932
4	71 %	364	1.000	0.900	0.806	0.937

Each row summarizes the average over all 38 ligands for the corresponding strategy

The results in Table 2 show more detailed results for each ligand with strategy 2. While there is some variation among the inhibitors, the coverage is almost always very high. In cases where it is not, such as AST-487, JNJ-7706621 and Sunitinib, it is usually a inhibitor that binds to many different parts of the kinome tree (see kinome interaction maps in [27]). Finally, we analyzed the sensitivity to the parameter *δ* and *λ*. As is shown in Tables 3 and 4, performance varies significantly with both *λ* and *δ* (as is expected). However, even with very large values of *δ*, the algorithm is still able to cover the vast majority of known binding affinities. Even more surprisingly, even when restricting SDPs to only *λ*=3 residues (corresponding to a *single* clustering), over 60 % of the structures with known binding affinity are covered.

**Table 2:** Coverage of labeled structures, number of predicted affinities for unlabeled structures, as well as specificity, precision, and accuracy for HPC-based prediction of binding affinity as recovered from SDPs computed using our algorithm (with *λ*=6 and *δ*=16). Sensitivity is equal to 1 in all cases

Inhibitor	Cov.	#pred.	Spec.	Prec.	Acc.
ABT-869	86 %	557	0.922	0.633	0.931
AMG-706	83 %	558	0.928	0.707	0.938
AST-487	65 %	426	0.661	0.806	0.859
AZD-1152HQPA	85 %	568	0.914	0.668	0.927
BIRB-796	67 %	391	0.766	0.653	0.838
BMS-387032/SNS-032	96 %	670	0.984	0.959	0.988
CHIR-258/TKI-258	81 %	420	0.947	0.861	0.960
CHIR-265/RAF265	87 %	473	0.960	0.801	0.966
CI-1033	77 %	475	0.882	0.710	0.909
CP-690550	96 %	629	0.989	0.736	0.989
CP-724714	99 %	684	0.999	0.982	0.999
Dasatinib	83 %	500	0.897	0.837	0.933
EKB-569	70 %	474	0.876	0.688	0.902
Erlotinib	80 %	532	0.902	0.693	0.920
Flavopiridol	80 %	515	0.844	0.754	0.895
GW-2580	99 %	677	1.000	1.000	1.000
GW-786034	79 %	485	0.920	0.737	0.934
Gefitinib	81 %	470	0.906	0.561	0.916
Imatinib	86 %	587	0.936	0.590	0.941
JNJ-7706621	59 %	356	0.580	0.704	0.790
LY-333531	83 %	413	0.912	0.652	0.924
Lapatinib	99 %	684	0.999	0.982	0.999
MLN-518	94 %	659	0.989	0.808	0.989
MLN-8054	87 %	493	0.948	0.766	0.956
PI-103	99 %	654	0.999	0.988	0.999
PKC-412	54 %	217	0.621	0.687	0.793
PTK-787	97 %	664	0.999	0.974	0.999
Roscovitine/CYC202	98 %	650	1.000	1.000	1.000
SB-202190	84 %	500	0.929	0.815	0.946
SB-203580	69 %	349	0.792	0.641	0.849
SB-431542	100 %	670	1.000	1.000	1.000
SU-14813	71 %	343	0.761	0.667	0.838
Sorafenib	70 %	509	0.919	0.801	0.939
Staurosporine	91 %	646	0.681	0.956	0.959
Sunitinib	61 %	343	0.652	0.654	0.790
VX-680/MK-0457	78 %	410	0.844	0.767	0.897
VX-745	85 %	583	0.912	0.680	0.926
ZD-6474	87 %	511	0.939	0.823	0.952
**average**	**83 %**	**520**	**0.887**	**0.783**	**0.929**

The last row lists the average performance over all inhibitors

**Table 3:** Sensitivity to the value of *λ* with *δ*=16

***λ***	Cov.	#pred.	Spec.	Prec.	Acc.
3	62 %	312	0.669	0.493	0.778
4	73 %	419	0.781	0.661	0.864
5	79 %	482	0.844	0.729	0.907
6	83 %	520	0.887	0.783	0.929
7	86 %	537	0.909	0.810	0.943
8	88 %	554	0.921	0.838	0.951
9	89 %	565	0.930	0.858	0.958

Each row represents an average over all 38 inhibitors

**Table 4:** Sensitivity to the value of *δ* with *λ*=6

***δ***	Cov.	#pred.	Spec.	Prec.	Acc.
1	85 %	587	0.904	0.820	0.941
2	85 %	580	0.903	0.817	0.940
4	85 %	565	0.900	0.812	0.938
8	84 %	547	0.895	0.800	0.935
16	83 %	520	0.887	0.783	0.929
32	81 %	490	0.871	0.723	0.916
64	78 %	456	0.848	0.658	0.898
128	74 %	413	0.817	0.612	0.876

Each row represents an average over all 38 inhibitors

## Discussion

### Frequency analysis of SDP positions

**Fig. 4:**
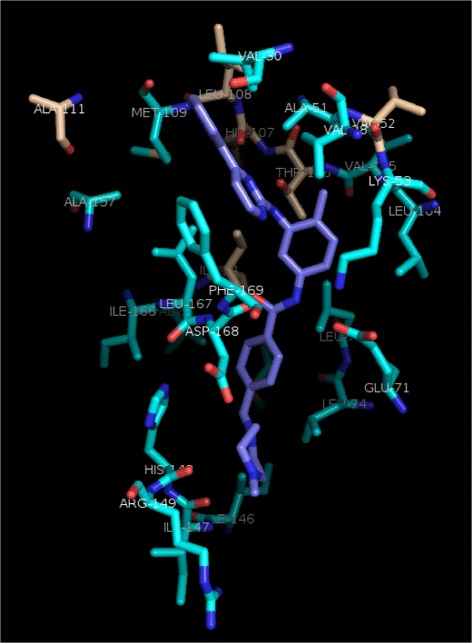
The kinase binding site. Selected residues of P38 *α* are shown in complex with imatinib (PDB ID 3HEC)

**Fig. 5:**
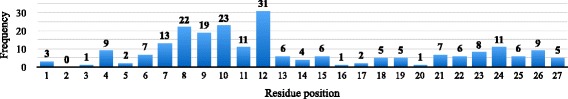
Frequency of each residue position occurring in SDPs across all inhibitors. The *x*-axis represents the residue position in the 27-residue multiple sequence alignment of the binding site

The 27 residues that make up the binding site (see Fig. 4) are not equally represented in the SDP profiles. For example, position 2 does not occur in any of the SDP profiles, whereas position 12 occurs in 31 out of the 38 (see Fig. 5). The residues occurring most frequently in SDP profiles are often residues that have been observed to be important for inhibitor selectivity.

SDP position 8, which occurs in 22 of the SDP profiles, corresponds to the well-known “gatekeeper” residue [28]. The size of this residue controls access to the hydrophobic binding pocket accessed by Type II inhibitors.

Most kinase inhibitors are ATP-competitive and mimic to a greater or lesser extent the hydrogen bonding interactions that the adenine aromatic moiety of ATP makes with the hinge region of the protein. The hinge region corresponds to positions 9–11 and each of these positions occurs frequently in the SDP profiles, particularly at positions 9 and 10. Note that the interactions of inhibitors with the hinge are through hydrogen bonds to the protein backbone and are thus, in this sense, not sequence specific. Also, position 10 is rarely involved in hydrogen bonding because the canonical orientation of the backbone orients the NH and CO backbone groups away from the binding site. A recent analysis has shown that the potency of kinase inhibitors is not correlated with the number of hinge hydrogen bonds, but that there is a trend, albeit not pronounced, for compounds that make more hydrogen bonds to be less selective [29]. Large conformational changes that alter the canonical binding pattern have been observed when the conformationally less constrained glycine residue occurs in hinge positions. The SDP analysis indicates that subtler alterations in geometry and sequence in this region play an important role in modulating selectivity. It is not inevitable that frequently observed interactions automatically translate into modulators of binding profile. Position 20 of the SDP profile corresponds to a conserved glutamic acid residue in the middle of the C-helix that forms a salt bridge with a conserved lysine and is often involved in hydrogen bonds to amides or ureas of Type II kinase inhibitors. However, this position occurs in only one SDP (SB-431542).

The most frequently selected position in SDP profiles is number 12, occurring in 31 out of the 38 profiles. This residue occurs in the “selectivity surface”, a relatively solvent exposed region with significant structural variation. For many inhibitors, this position contributes information from multiple 3-residue subsets, enabling the geometric and sequence variability of this region of the protein relative to the rest of the structure to be captured.

Positions 16 and 17 correspond to the Asp and Phe residues of the DFG motif. This motif occurs in “DFG-in” or “DFG-out” conformations, with DFG-in being the active conformation of the enzyme and DFG-out a catalytically inactive form that is stabilized by Type-II inhibitors such as imatinib. Despite this geometrical variability, these positions occur rarely in SDP profiles. Only a small percentage of kinases have been observed in the DFG-out state crystallographically. Interestingly, the ability of a kinase to adopt this inactive conformation has been postulated to be controlled by two other residues, the gatekeeper and the residue immediately N-terminal to the DFG sequence [30]. This later residue is at position 15 and occurs in the SDPs with moderate frequency.

**Fig. 6:**
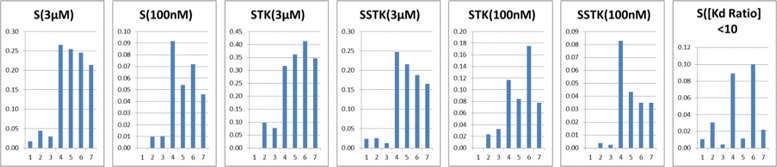
Different measures of selectivity as a function of the number of 3-position subsets that contribute to the SDP profile

The number of 3-position subsets that contribute to the SDP profile is related to inhibitor selectivity. The histograms in Fig. 6 show the number of contributing 3-position subsets (*x*-axis) plotted against the various selectivity metrics calculated by Karaman et al. [27] (*y*-axis). The selectivity values are the average of the compound values with SDP profiles derived from that number of 3-position subsets. Note that the selectivity value can be zero. For all metrics other than the *K*_d_ ratio measure, the most selective inhibitors have SDP profiles derived from one to three 3-position subsets. The pattern is similar whether the kinases are considered as a whole (S(3 *μ*M), S(100nM)) or the tyrosine kinases (STK(3 *μ*M), STK(100nM)) or serine/threonine kinases (SSTK(3 *μ*M), SSTK(100nM)) are considered separately. A very similar result is obtained by calculating S(10 *μ*M) from the Karaman et al. data [27], in order to match the activity cutoff threshold used in the CCORPS analysis (data not shown). The *K*_d_ ratio measure differs from the others by focusing on off-targets with affinity within 10-fold of the primary target. Such compounds are considered active by the 10 *μ*M IC50 cutoff value used to generate the SDPs and thus the lack of correlation with the *K*_d_ ratio measure is expected. A similar trend is observed in specificity of the SDP profiles. In Table 5 we see that SDP profiles derived from a small number of 3-position subsets tend to a higher specificity.

**Table 5:** Average specificity over all inhibitors as a function of the number of 3-position subsets that determine the SDPs

# 3-pos. subsets	1	2	3	4	5	6	7
Specificity	1.00	0.99	0.97	0.83	0.86	0.86	0.83
Frequency	1	6	4	10	7	6	4

The last row shows the number of inhibitors whose sdps are determined by a given number of 3-position subsets

### Comments on specific compounds

#### CP-690550 (Tofacitinib)

**Fig. 7:**
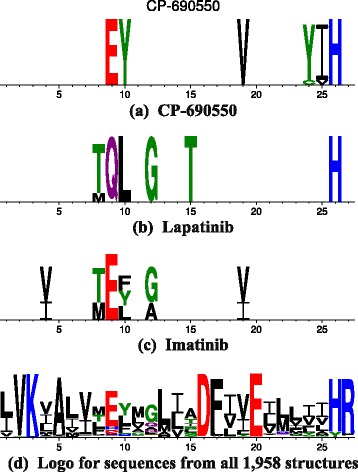
Sequence logos (created by WebLogo [38]) for the SDPs of structures known to bind to different inhibitors as well as a logo for all structures

Tofacitinib is a clinically used selective Janus Kinase inhibitor. An SDP Word Logo is shown in Fig. 7a.

There are PDB structures for 5 kinases, for each of which tofacitinib is a potent inhibitor (JAK1, JAK3, JAK3, TYK2 and PKN1).

In the X-ray structure 3lxk (JAK3) elements 9, 10 and 19 are close to the inhibitor, but elements 24–26 are distant. Figure 1 shows that this arises from two 3-position subsets (9, 10, 26 and 19, 24, 25) [This being the case, I’m not sure why there is variability at positions 24 and 25].

The tofacitinib complexes with JAK1, JAK2, JAK3 and TYK2 are very similar to each other. The structure 4oti is the PKN1-tofacitinib complex, for which tofacitinib is a medium potency inhibitor. Superposition of the ligand between 3lxk and 4oti shows an essentially identical conformation. This aligns the residues of the N-lobe quite well, but the C-lobe is displaced. The 3-position subsets that span the N- and C-lobes could capture this range of possibilities in HPCs and thus enable the binding to PKN1 to be accounted for. Positions 24–26 occur quite frequently as SDPs, even for inhibitors that are not in contact with these residues.

The p38 *α* structure (e.g., 3hec) is not inhibited by tofacitinib. The superposition (based on the 27 alpha-carbon positions of the binding site residues used by CCORPS) shows a broadly similar disposition of the N- and C-lobes. In this case there are sequence differences at five of the six SDP positions. The CDK8 structure (3rdf) has more subtle differences that are hard to distinguish from the active examples based on visual inspection.

Weigert et al. [31] generated resistance mutants to JAK2. Of the three mutants identified, one E864K (JAK2 numbering) is not within our 27 residue active site definition. However, Y931C (Position 10 in the logo) conferred resistance to all of the JAK inhibitors studied, including tofacitinib, in agreement with the SDP result. G935R (Position 12 in the logo) conferred resistance to all inhibitors except tofacitinib, also in agreement with the SDP.

#### Lapatinib

Lapatinib is a selective inhibitor of ErbB2 and EGFR. An SDP Word Logo is shown in Fig. 7b.

The general pattern is fairly typical, with the gatekeeper, hinge and selectivity surface represented. Kancha et al. [32] reports several mutations observed in ERBb2 in various solid tumors. Most of these are distant from the binding site, but one T862A corresponds to position 15 in the Logo and is associated with modest lapatinib resistance. An analogous mutation is also found in EGFR.

Trowe et al. [33] report that T798 is the most frequently mutated ErbB2 residue in an in vitro screen using a randomly mutagenized ErbB2 expression library and shows the greatest lapatinib resistance. This corresponds to position 8 in the logo (gatekeeper). A less frequently observed mutation L726 is not found in the logo (position 1). Other mutated residues are not in the binding site set.

The gatekeeper residue is also mutated in EGFR, but other EGFR resistance-inducing mutations do not map to the corresponding logo positions.

#### Imatinib (Gleevec)

Imatinib is an Abl/Kit/VEGFR inhibitor. An SDP Word Logo is shown in Fig. 7c.

The profile is similar to that of lapatinib to the extent that gatekeeper, hinge and selectivity surface residues are represented. Mutation at positions 8 or 10 is a common cause of imatinib resistance. Note that the presence of the gatekeeper in the profile of a Type II inhibitor is not unexpected, but that not all Type-II logos have this. As noted above, Type II inhibitors such as imatinib bind to a DFG-out enzyme conformation, but these residues are not in the profile and thus do not provide the strongest selectivity signal.

Position 19, which is in the hydrophobic pocket, is also of interest. Mutation at this position in BCR/ABL has been reported to confer moderate Imatinib resistance [34]. This position was also the most frequently mutated residue found in imatinib-resistant KIT mutants from analysis of tumor samples obtained from patients enrolled in a Phase II clinical study of imatinib [35]. The gatekeeper residue was also frequently mutated in this population. Sunitinib (Sutent™) is approved for the treatment of advanced GIST after failure of imatinib due to resistance or intolerance. It is effective against the imatinib-resistant V654A (position 19) mutant, a position which does not occur in the sunitinib SDPs.

If false-HPCs are omitted (i.e., strategy 3 in the subsection *Coverage and Predictive Power of*SDP*s*), the SDPs also include position 24. This position is frequently mutated in resistant tumors, with positions 10 and 24 together accounting for 14 % of BCR/ABL mutations. The SDPs of the more selective KIT/VEGFR inhibitor PTK-787 also includes position 24.

The occurrence of other positions in the imatinib logo is harder to rationalize. In the structures, the side chain at position 4 points away from the inhibitor and is not in direct contact with it. This may point to an indirect role in modulating the conformation of the protein in this region. Position 4 is actually selected quite frequently (9 times). As part of the hydrophobic core of the N-lobe, it may act as a marker for the relative disposition of the two domains of the enzyme. Differential flexibility of the kinases is often discussed in the literature as playing a role in selectivity, see for example [36].

## Conclusion

We have described a general method for identifying Specificity Determining Positions in families of related proteins. The method was shown to be very effective in identifying SDPs within the human kinome that help explain the binding affinity of 38 different inhibitors. Consistent with prior studies, we were able to identify the gatekeeper residue and the hinge region as generally very important for the binding specificity of kinases. It has also highlighted the selectivity surface as a region that is key in determining selectivity profiles. An in-depth analysis of the SDPs for three specific kinase inhibitors provides further evidence that we can identify other residues that are known to be important in each case, including positions that are mutated in drug-resistant tumors. Of particularly interest are these that are not in direct contact with the inhibitor (some examples of which were discussed above) but which may be involved indirectly through, for example, influencing the conformation or flexibility of the protein. This would be a significant benefit, as such residues are difficult to identify by other means. Not only could this potentially provide a new insight into the structural biology of kinases, but such knowledge may be helpful in the design of inhibitors with novel, or improved, selectivity profiles. In this regard, it would be interesting to explore expanding the approach to include additional, non-binding site residues, that have been implicated in resistance through modulation of conformational plasticity and investigated by molecular dynamics.

In prior work [37] we have demonstrated that the addition of homology models leads to an improvement in the prediction of binding affinity. Homology models can fill in gaps in structural coverage, thereby potentially eliminating “accidental” HPCs and create new ones. In future work we plan to investigate whether homology models can provide similar benefits in the identifications of SDPs.

## Abbreviations

CCORPS, combinatorial clustering of residue position subsets; HPC, highly-predictive cluster; SDP, specificity-determining position.
